# Dynamic analysis of the microbial communities and metabolome of healthy banana rhizosphere soil during one growth cycle

**DOI:** 10.7717/peerj.14404

**Published:** 2022-11-18

**Authors:** Liujian Ye, Xiaohu Wang, Shengbo Wei, Qixia Zhu, Shuang He, Liqin Zhou

**Affiliations:** 1 Guangxi Biological Science and Technology Research Center, Guangxi Academy of Sciences, Nanning, China; 2 State Key Laboratory of Non-Food Biomass and Enzyme Technology, Guangxi Academy of Sciences, Nanning, China; 3 National Engineering Research Center for Non-Food Biorefinery, Guangxi Academy of Sciences, Nanning, China

**Keywords:** Healthy banana planting, Soil microbial diversity, Soil metabolomics, Soil microecology, Microbial interactions

## Abstract

**Background:**

The banana-growing rhizosphere soil ecosystem is very complex and consists of an entangled network of interactions between banana plants, microbes and soil, so identifying key components in banana production is difficult. Most of the previous studies on these interactions ignore the role of the banana plant. At present, there is no research on the the micro-ecological environment of the banana planting growth cycle.

**Methods:**

Based on high-throughput sequencing technology and metabolomics technology, this study analyzed the rhizosphere soil microbial community and metabolic dynamics of healthy banana plants during one growth cycle.

**Results:**

Assessing the microbial community composition of healthy banana rhizosphere soil, we found that the bacteria with the highest levels were Proteobacteria, Chloroflexi, and Acidobacteria, and the dominant fungi were Ascomycota, Basidiomycota, and Mortierellomycota. The metabolite profile of healthy banana rhizosphere soil showed that sugars, lipids and organic acids were the most abundant, accounting for about 50% of the total metabolites. The correlation network between fungi and metabolites was more complex than that of bacteria and metabolites. In a soil environment with acidic pH, bacterial genera showed a significant negative correlation with pH value, while fungal genera showed no significant negative correlation with pH value. The network interactions between bacteria, between fungi, and between bacteria and fungi were all positively correlated.

**Conclusions:**

Healthy banana rhizosphere soil not only has a stable micro-ecology, but also has stable metabolic characteristics. The microorganisms in healthy banana rhizosphere soil have mutually beneficial rather than competitive relationships.

## Introduction

An agroecosystem refers to both the agricultural biological population and the agroecological environment. Agroecosystems are self-organizing systems. Different elements of the system, such as crops, microorganisms, and soil, interact with each other and respond to different management practices, but at the system level, these interactions have been largely unexplored (Ray et al., 2020). From a biological and physicochemical standpoint, soil is the most diverse ecosystem on Earth, harboring a wide variety of organisms, many of which are still unknown. Microbial activity affects soil structure in many ways: from long-term increases in global soil respiration and associated soil carbon losses that lead to degraded soil structure, water erosion, and susceptibility to wind erosion (Davidson, Savage & Finzi, 2014), to surface-adherent microorganisms that promote mineral weathering affecting soil colonies (Goudie & Viles, 2012). Microbial interactions within the rhizosphere greatly affect the soil physical environment in a short time frame (Philippot et al., 2013). The structure, function, interactions, and dynamics of microorganisms that form complex communities are critical to agroecosystems. They are the most diverse biota on Earth and play an integral role in keeping the ecosystem functioning and in the biogeochemical cycling of carbon, nitrogen, sulfur, phosphorus, and metals (Kamal, Prasad & Varma, 2010). The oldest microorganisms in soil are bacteria and archaea, which have been observed in many different environments. Fungi appeared relatively late, and it is thought that terrestrial fungi may have co-evolved with plants because the two are so closely related. Agricultural management practices can also have a significant impact on plant-microbe interactions.

The root zone of crops is an important soil microhabitat, and there are extensive and complex biological associations between microorganisms and root cells (Kuzyakov & Razavi, 2019). The characteristics of plant species greatly affect the various organisms that live in the soil, especially the microorganisms that live near the rhizosphere of plants, which release a variety of chemicals that can recruit or prevent certain microbial taxa from forming-specific microbial assemblages in the root zone (Jacoby & Kopriva, 2019; Reinhold-Hurek et al., 2015). In turn, the microbial associations in the rhizosphere can significantly affect plant fitness and soil biophysical and chemical properties, which can affect plant development and growth (Debenport et al., 2015; Jones et al., 2019; Liu et al., 2017; van der Putten et al., 2013). To establish a symbiotic relationship with plants, microorganisms release many beneficial compounds in the rhizosphere for plant uptake, and these molecules facilitate the regulation of plant transcriptomes. Microbial populations near plant roots produce plant hormones and secrete some cytokinins, auxins, and gibberellins (Fahad et al., 2015). Plant-specific root exudates also impact the rhizosphere microbial communities; for example, fumaric acid secreted by banana roots attracts *Bacillus subtilis* to the roots, which then form biofilms in the rhizosphere (Zhang et al., 2013). Some compounds have been shown to induce nodulation, such as flavonoids, which are derivatives of 2-phenyl-1, 4-benzopyrone. These activate bacterial nodulation genes leading, to lipochitooligosaccharide, which triggers nodulation. These compounds have a taxonomic role in mimicking bacterial quorum sensing, thus affecting bacterial metabolism (Hassan & Mathesius, 2012). In addition, several other compounds contribute to the synthesis of the phytohormones needed by bacteria to promote rhizobacterial activity in plant growth, such as tryptophan for the biosynthesis of indoleacetic acid (Haichar et al., 2014). Aminocyclopropane-1-carboxylic acid is also secreted by roots for the synthesis of ethylene (a stress hormone) and also serves as a carbon and nitrogen source for bacterial growth (Haichar, Roncato & Achouak, 2012). Plant-derived metabolites may be important factors affecting the structure of the rhizosphere soil microbiome, as metabolites can determine microbial food webs, regulate soil chemistry, alter microbial gene expression, and even act as semiochemicals that mediate microbial-microbe interactions (Hu et al., 2018; Voges et al., 2019). Liquid chromatography-mass spectrometry (LC-MS) is an analytical chemistry technique that combines the physical separation capability of high-performance liquid chromatography (LC) technology with the mass analysis capability of mass spectrometry (MS). Liquid chromatography technology separates mixtures from various components, and mass spectrometry technology is responsible for detecting and analyzing the structural characteristics of each component with polymer specificity and detection sensitivity. The two technologies have been synergistically enhanced in LC-MS technology, which has been widely used in soil metabolomics. Combining the composition of soil rhizosphere microbial diversity and soil metabolite composition can provide a deeper understanding of the complex relationships in rhizosphere soil (Morton et al., 2019).

Bananas are a perennial crop that grow quickly and can be harvested once a year. They are the largest monoculture crop in the world (Baset Mia, Shamsuddin & Mahmood, 2010; Mia et al., 2010), are an important economic and food crop in the tropics and subtropics, and are one of the top ten staple foods in the world (Butler, 2013). Cultivated bananas propagated through asexual reproduction are mostly susceptible to various pests and diseases, especially banana wilt, which is one of the major constraints to banana production worldwide. Banana wilt is caused by soil-borne fungus *Fusarium oxysporum* F, which infects the xylem through the roots of the banana plants, causing extensive necrosis, leading to the wilting of the banana plants (Dita et al., 2018). The current methods for preventing and controlling banana fusarium wilt are crop rotation, selection of disease-resistant varieties, and chemical or biological methods, but these methods are not very effective, and the disease is still spreading between continents, countries, and regions (Getha & Vikineswary, 2002; Wang et al., 2012). In general, biological control is considered a safe, environmentally friendly, and cost-effective method of disease control (Huang et al., 2017; van Bruggen et al., 2015). The banana growing ecosystem is very complex and consists of an entangled network of interactions between banana plants, microbes and soil, so identifying key components in banana production will be important to understanding these interactions. Most of the current research on the biological control of banana plant diseases is based on comparing the differences in the microbial communities of disease-free and diseased soils at a certain point in time, or screening certain probiotics that may help prevent banana fusarium wilt and when applied to the soil, but these studies often ignore the role of the banana plants in the soil. The overall change in the cycle of the soil micro-ecological environment may be one of the key factors in explaining why the current methods of biological control are not very effective in promoting the healthy development of banana plants. Therefore, this article studies and analyzes the dynamic changes of the rhizosphere soil microbial community and metabolism during one growth cycle of healthy banana plants, in order to provide an experimental reference for future banana planting ecosystem research as well as research on soil microorganisms in agro-ecosystems.

## Materials and Methods

### Field planting

The tracking and sampling location used in this study is located in Nanxu Town, Long’an County, Nanning City, Guangxi, one of the main areas of banana production in China (E107°39′0″ N23°5′19″). This area has an altitude of 232.3 m, is located south of the Tropic of Cancer, and has a subtropical monsoon climate. The territory is humid and rainy, with concentrated rainfall and abundant sunshine. It is more hot than cold, experiences little wind, rarely has snow and ice, and only sees the occasional severe frost. The average annual rainfall is 1,301 mm. The distribution of rainfall during the year is uneven, with strong seasonality. The rainfall is mostly concentrated in the summer, and the annual average temperature is 21.8 °C. The selected banana planting area for this study was the 20-hectare banana planting base of Najintian Agricultural Investment Co., Ltd. in Longan County, Guangxi, China. The banana tissue seedlings (*Musa acuminata* AAA *Cavendish cv*. Brazil) were planted in March 2020 with a planting density of 120–130 trees/667 m^2^ and were managed according to standard agricultural practices. In order to understand the background of soil microbial communities and metabolic changes during a banana planting cycle in Guangxi, this study started the first sampling in April 2020 (1 month after banana planting). The soil from the rhizosphere of 10 banana plants was collected, and marks were made on the chosen banana trees, so that the same 10 trees could be continuously tracked. Soil samples were collected at regular, 1 month intervals, and changes in the soil microbial community as well as metabolic changes during the growth cycle from banana seedlings to long fruit harvesting were continuously tracked. Ten soil samples were taken at each of the sample collection periods for a total of 70 samples. The collection periods were recorded as: M1 (April), M2 (May), M3 (June), M4 (July), M5 (August), M6 (September), and M7 (October). The 10 banana plants that were tracked in this study were all healthy and free of blight.

### Soil sample pretreatment and determination of pH and organic matter

Soil samples were transported on ice to our laboratory. One part of each soil sample was air-dried and sieved to 2 mm for a chemical property analysis, and the other part of the soil was stored at −80 °C for subsequent experiments. The methods used for measuring the basic chemical properties of the soil were those outlined in previous studies (Xiong et al., 2015). Briefly, soil pH was measured with a glass electrode meter at a soil-water ratio of 1:2.5 (w/v). Soil organic matter was determined using the potassium dichromate external heating method.

### Soil DNA extraction, library construction, and sequencing

The MN NucleoSpin 96 Soil DNA extraction kit was used according to the manufacturer’s instructions for the extraction of total genomic DNA from the soil. A two-step library construction method was used for microbial diversity library construction and sequencing he first step uses DNA as a template to design primers with adapters for PCR, and the second step uses the PCR product of the first step as a template for PCR. The purpose of primer adapters is to facilitate adding a barcode/index in the second step of library construction to distinguish the base sequence of the sample (Miya et al., 2015). The bacterial 16S V3+V4 region were used to amplify the primers 338F 5′-ACTCCTACGGGAGGCAGCA-3′ and 806R 5′-GGACTACHVGGGTWTCTAAT-3′. The fungal ITS region were used to amplify the primers ITS1F 5′-CTTGGTTCATTTAGAGGAAGTAA-3′ and ITS2 5′-GCTGCGTTCTTCATCGATGC-3′. All amplifications were performed in 10 μL systems, including genomic DNA 50 ng, 0.3 μL each of 10 μM forward and reverse primers, KOD FX Neo Buffer 5 μL, dNTPs (2 mM each) 2 μL, KOD FX Neo Enzyme 0.2 μL, and ddH2O supplemented to 10 μL. PCR conditions were 95 °C for 5 min, 95 °C for 30 s, 50 °C for 30 s, 72 °C for 40 s, followed by 25 cycles, a final extension at 72 °C for 7 min, then held at 4 °C. PCR products were mixed at a mass ratio of 1:1 according to the results of electrophoresis quantification (ImageJ software). After mixing, the samples were purified by the OMEGA DNA purification column. After electrophoresis with a 1.8% agarose gel at 120 V for 40 min, the target fragments were cut and recovered to form a sequencing library. The constructed library was first subjected to library quality inspection, and the library that passed the quality inspection was sequenced by Illumina HiSeq 2500. All library preparations were performed on the Illumina Sequencing platform of Beijing Biomarker Technologies.

### Sequence data processing and analysis

Trimmomatic (version 0.33) was used to filter the quality of raw data, Cutadapt (version 1.9.1) was used to identify and remove primer sequences, and then USEARCH (version 10) was used to splice paired-end reads and remove chimeras (UCHIME, version 8.1), resulting in high-quality sequences for subsequent analysis. Sequences were clustered at the 97% similarity level (USEARCH, version 10.0) and OTUs were filtered with a threshold of 0.005%. UNITE was used as the reference database and the naive Bayes classifier was used to annotate the feature sequence taxonomically. After the species classification information corresponding to each feature was obtained, the QIIME software counted the composition of each sample community at each level (phylum, genus). The species abundance table was then drawn into the community structure map of the samples at each taxonomic level using the R language tool. A Shannon index analysis was performed using the Mothur (version v. 1.30) software, and then a Spearman rank correlation analysis was performed on the sequencing results. The data with *p* values less than 0.05 were screened to construct a correlation network and create a correlation heatmap based on R heatmap. All sequencing data analyses were performed on the BMKCloud platform.

### Extraction, detection and analysis of metabolites

The machine analysis by untargeted LC-MS/MS refers to the method of Zhu et al. (2022). All metabolite data analysis was performed on the BMKCloud platform.

### Statistical analysis

Statistical analysis of the data refers to the method of Shen et al. (2015). The distributions of bacterial and fungal species in the two types of soil samples were compared using the Wilcoxon rank-sum test in the SPSS software.

## Results

### Community distribution of banana rhizosphere soil microorganisms

**Figure 1:**
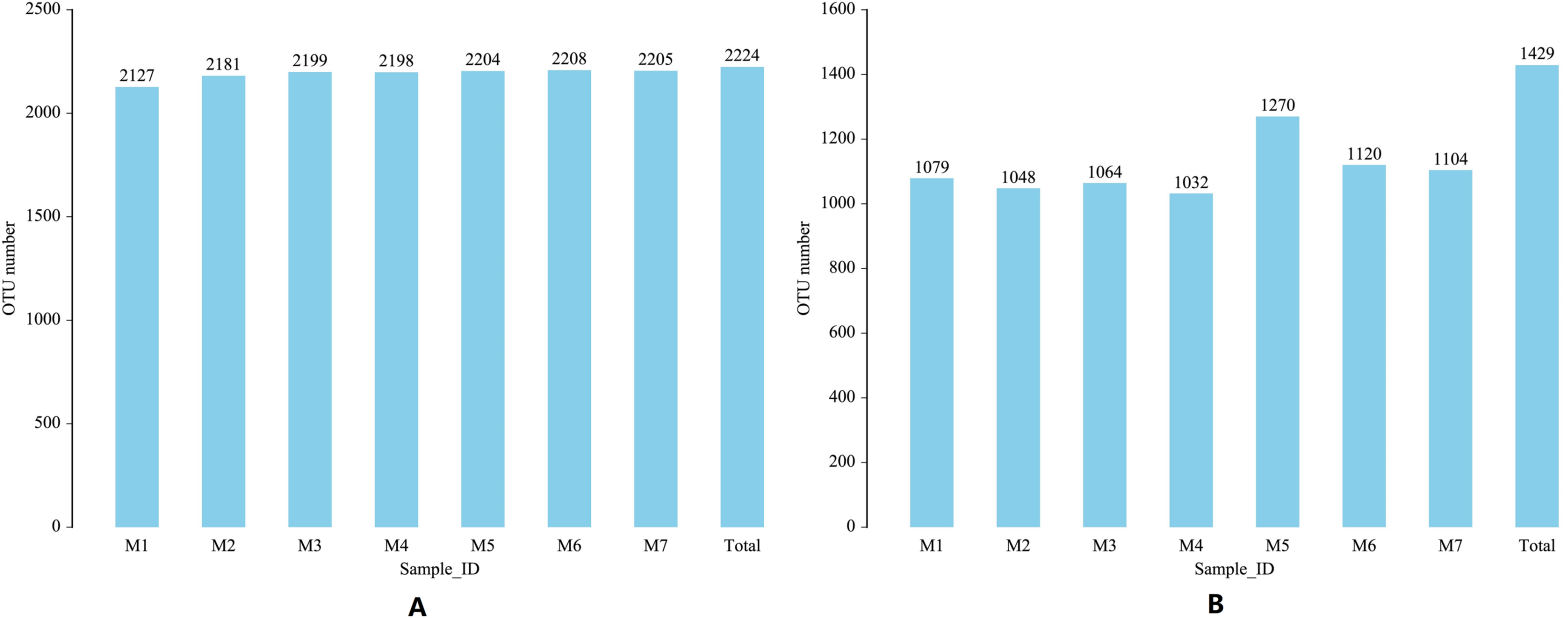
Changes in the soil microbial OTU communityduring one growth cycle. (A) Distribution map of number of bacterial OTUs. (B) Distribution map of number of fungal OTUs. The abscissa M1-M7 represents soil samples in different planting periods, the ordinate is the number of OTUs, and the number above the column is the number of OTUs of the corresponding sample. “Total” indicates the total number of all OTUs in a growth cycle.

The 16S region and ITS region of 70 banana rhizosphere soil samples were sequenced, and a total of 5,603,074 pairs of 16S region reads and 5,598,535 pairs of ITS region reads were obtained. After quality control and splicing of paired-end reads, 5,575,880 16S region clean reads and 5,578,734 ITS zone clean reads were generated. Based on clustering at the 97% similarity level, a total of 2,224 16S rRNA gene OTUs and 1,429 ITS gene OTUs were obtained from all samples. Over the entire banana planting growth cycle, the number of bacterial OTUs first increased and then decreased and these differences were statistically significant (F = 2.6335, *p* = 0.0242). Early in the planting cycle, there was a small range in soil fungal OTUs, with these levels suddenly rising and then falling in the middle phases of the growth cycle (F = 3.7899, *p* = 0.0028). These results show that both the bacterial community and fungal community experience, a certain range of dynamic changes during the banana growth cycle (Fig. 1).

**Figure 2:**
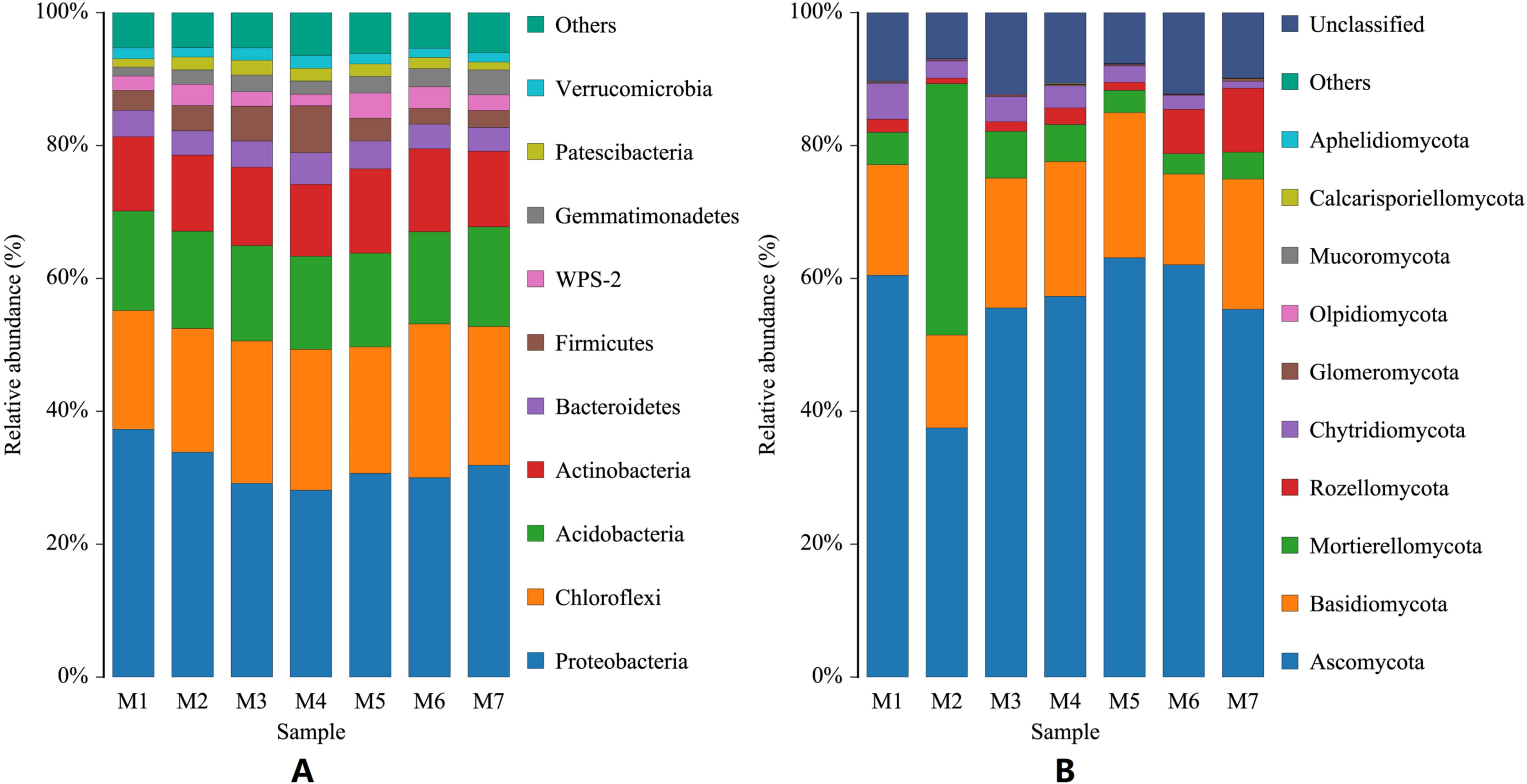
Histogram of phylum-level distribution of microbial species in banana rhizosphere soil. (A) Histogram of bacterial species distribution at the phylum level. (B) Histogram of fungal species distribution at the phylum level. The abscissa M1–M7 represents soil samples at different planting periods, and the ordinate is the relative abundance percentage of each species. The color blocks represents a species, the individual species, with each species being represented by a different color, and the length of the color block representing the relative abundance ratio of the species; for visual simplicity, only the abundance levels of the top 10 species are displayed, with other species identified merged into others. Unclassified represents that are not yet taxonomically annotated.

During the growth cycle of banana planting, the composition of the rhizosphere soil microbial community changed based on changes in the conditions of the growth environment. At the phylum level, changes in the number of bacterial communities were small, and the phyla with the highest levels were Proteobacteria, Chloroflexi, and Acidobacteria, whose annual average relative abundances were 31.57%, 20.312%, and 14.41%, respectively. With the passage of planting time, Proteobacteria first gradually decreased and then fluctuated slightly, with the lowest relative abundance in the M4 period (F = 2.3625, *p* = 0.0403); Chloroflexi gradually increased and then fluctuated slightly (F = 0.9793, *p* = 0.4468); while Acidobacteria remained relatively stable (F = 0.0825, *p* = 0.9977), with a relative abundance fluctuating around 14.41%. The Actinobacteria phylum had a relatively high relative abundance (F = 0.6244, *p* = 0.7100), which fluctuated in a small range around 11.70%. Compared with the bacterial community, the relative abundance of the fungal community experienced larger dynamic changes. The dominant fungal phyla were Ascomycota, Basidiomycota, and Mortierellomycota. Ascomycota declined significantly in the M2 period, then a rapidly increased in the M3 period, followed by a slow increase and then a slow decline (F = 4.1037, *p* = 0.0015). Fungal phyla Basidiomycota declined significantly in the M2 and M6 periods, and fluctuated less in the other periods (F = 0.77800, *p* = 0.5902). Mortierellomycota increased sharply in the M2 period, and fluctuated just a small range in the other periods (F = 22.4264, *p* = 6.0209E−14). In the later growth periods (M6 and M7 stages), the relative abundance of the Rozellomycota phylum increased slightly (F = 1.1785, *p* = 0.3292; Fig. 2).

**Figure 3:**
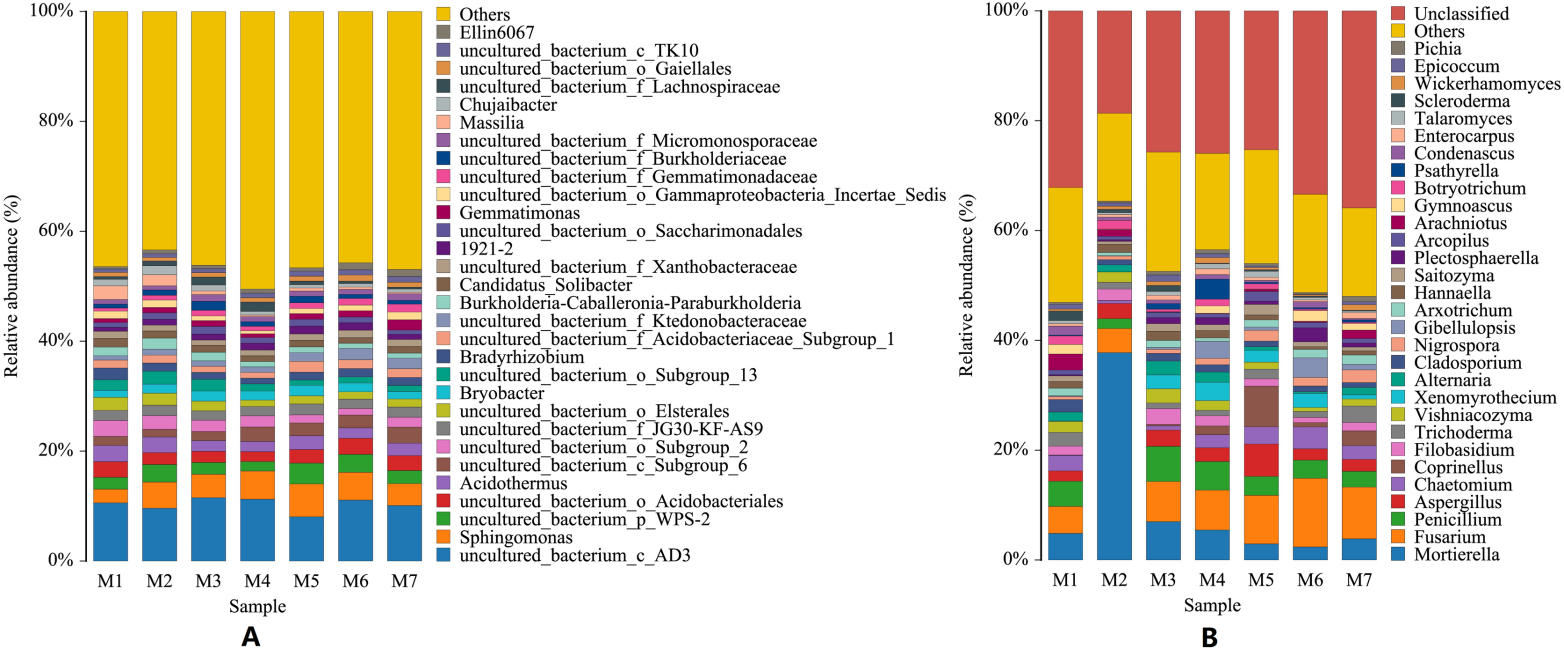
Histogram of genus-level distribution of microbial species in banana rhizosphere soil. (A) Histogram of bacterial genera species distribution. (B) Histogram of fungal genera species distribution. The abscissa and ordinate are the same as in Fig. 2. For visual simplicity only the abundance levels of the top 30 genera are displayed, with all other species identified merged into an “others” category.

At the genus level, the species of bacteria were widely distributed, and the diversity of soil bacteria was rich. The top three genera were uncultured_bacterium_c_AD3, *Sphingomonas*, uncultured_bacterium_p_WPS-2, and their average relative abundances were 10.32%, 4.52%, and 2.66%, respectively. Among the top 10 genera in relative abundance, 70% were uncultured bacteria. The dynamic changes seen at the bacterial genus level were smaller, with a wave-pattern of changes. The genus *Sphingomonas* was the lowest in the M1 period, and then remained generally stable (F = 3.2677, *p* = 0.0073). Compared with bacterial genera, the dynamic changes seen in fungal genera were larger, and the top 10 fungal genera were all culturable. The number of fungal genera species identified was similar to the number of bacterial genera species, but the fungal species distribution was wider, and the diversity of the soil fungi was also rich. The main fungal genera identified in the banana rhizosphere soil were *Mortierella*, *Fusarium* and *Penicillium*. *Fusarium* is the pathogen that has been identified as causing banana fusarium wilt. The relative abundance of *Fusarium* in the soil of this study varied from 4.37% to 12.50%, with an annual average relative abundance of 7.80%. The genus *Mortierella* showed a sudden surge in the M2 phase and then dropped sharply to a lower level (F = 22.8489, *p* = 4.07523E−14). The relative abundance of *Penicillium* first sharply decreased in the M2 stage, and then gradually decreased after a brief increase in the M3 stage (F = 1.2244, *p* = 0.3059). *Fusarium* continued to increase with time, reaching the highest levels in the M6 stage and then started to decline again in the M7 stage (F = 1.6136, *p* = 0.1581). Increases in the levels of *Fusarium* were correlated with decreases in the levels of both *Mortierella* and *Penicillium* (Fig. 3).

### Dynamic changes in the microbial diversity of the banana rhizosphere soil

**Figure 4:**
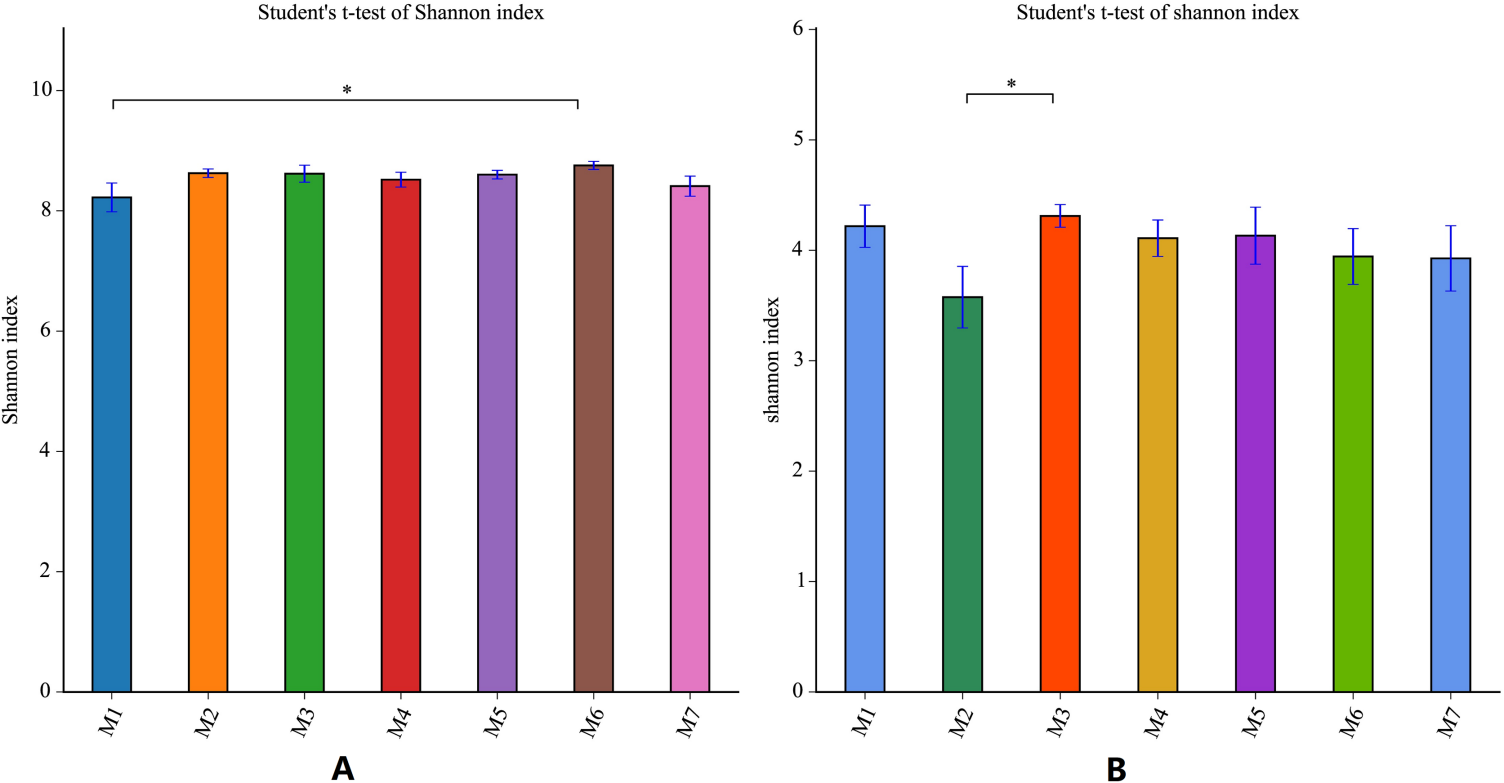
Shannon index analysis of banana rhizosphere soil microorganisms. (A) Bacterial Shannon Index of each planting period. (B) Fungal Shannon Index of each planting period. The Shannon index measure species diversity. The larger the index, the higher the community diversity. The abscissa and ordinate are the same as in Fig. 2. An asterisk (*) indicates a significant correlation at *p* ≤ 0.05.

During the one growth cycle of this study, the differences in the Shannon index of the banana soil bacteria and the soil fungi were not significant, indicating that the soil microbial diversity was stable, and the small fluctuations seen in each period also dynamic fluctuations in a steady state. The Shannon index of soil bacteria was the smallest in the M1 stage, and gradually increased with time, reaching the highest value in the M6 stage, and then decreasing again in the M7 stage. The difference between the Shannon index of M1 and the Shannon Index of M6 was the largest. This indicates that the bacterial diversity of the soil is smallest in the early stages of banana planting. The bacterial diversity increased steadily as the banana plants grow, with the M6 stage having the largest bacterial diversity (F = 1.5646, *p* = 0.1723). The Shannon index of fungi fluctuated more than the Shannon index of bacteria. The Shannon index of soil fungi started high in M1, dropped to the lowest in the M2 stage, rose to the highest level in the M3 stage, and then gradually decreased in the subsequent planting stages, but remained higher than in the M2 stage. This pattern means that the fungal diversity was the largest at the early stage of planting, decreased sharply in the M2 stage, then reached the highest level in the M3 stage (F = 6.1288, *p* = 0.0235) before decreasing again. Throughout the whole banana planting cycle, the bacterial diversity was relatively stable, while the fungal diversity fluctuated greatly (Fig. 4).

### Characteristics of and changes in the metabolites in banana rhizosphere soil

**Figure 5:**
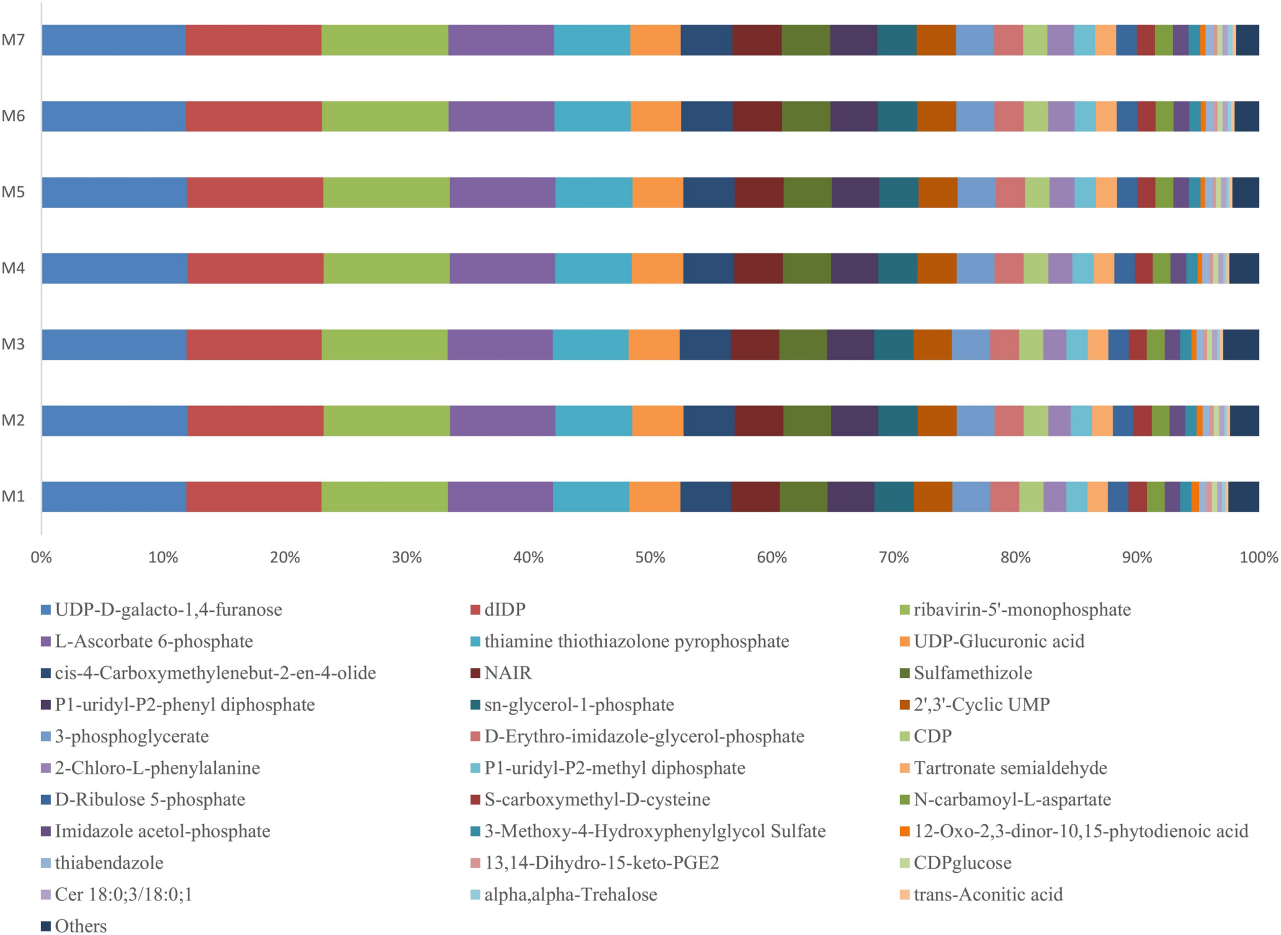
Profiles of the metabolites in the banana rhizosphere soils. The abscissa and ordinate are the same as in Fig. 2. This figure shows the difference comparison of the relative abundance of the top 30 metabolites, with the rest of the metabolites assigned to the “others” category.

During one growth cycle, we analyzed the characteristics of the metabolites in the banana rhizosphere soil and detected a total of 130 metabolites, including 17 sugars and their derivatives, 34 organic acids, eight bases and nucleosides, and 18 esters, eight alcohols and aldehydes, 13 amino acids, nine pesticides and biotin, six amines and derivatives, and five aromatic compounds. These metabolites were the most abundant in carbohydrates, lipids, and organic acids, accounting for about 50% of the total metabolites. Comparing the changes of the metabolites at different stages of banana growth showed that most of the metabolites in the soil were in a stable state. Among all the metabolites detected, the UDP-D-galacto-1, 4-furanose metabolite had the highest content, accounting for 12% of the total metabolite content, and it remained stable throughout the M1–M7 stages of the banana growth cycle (F = 1.6107, *p* = 0.1589; Fig. 5).

### Correlation analysis of banana rhizosphere soil pH, organic matter, and soil microorganisms

**Figure 6:**
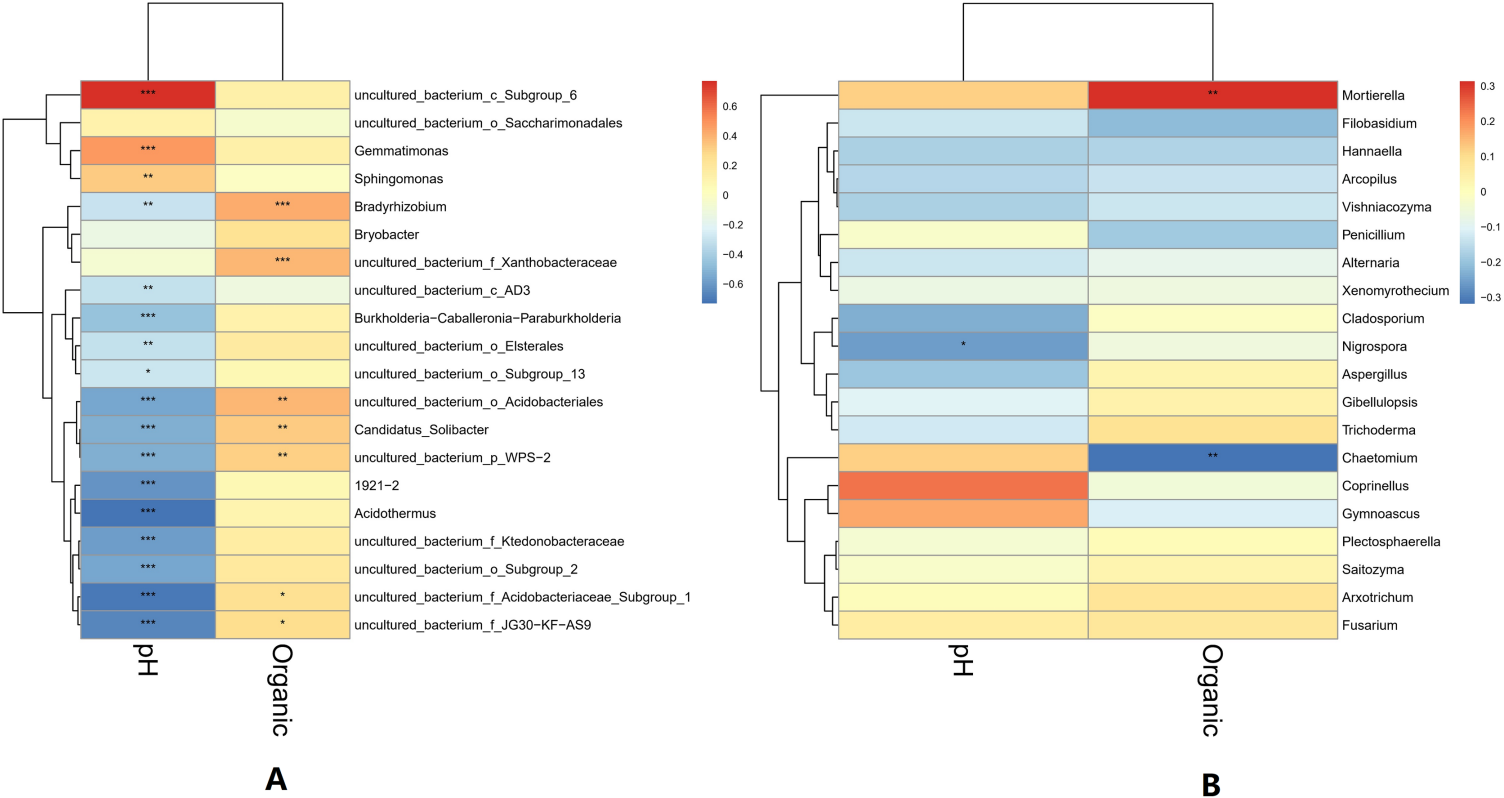
Heatmap of correlations between microbial genus levels and pH and organic matter. (A) Heatmap of correlations between bacterial genera and pH and organic matter. (B) Heatmap of correlations between fungal genera and pH and organic matter. The horizontal axis is pH and organic matter (labeled “Organic”), respectively, and the vertical axis is the microbial species at the genus level. The darkness of the color represents the strength of the correlation. Red represents positive correlation, and blue represents negative correlation. An asterisk (*) indicates *p* ≤ 0.05, significant correlation, Two asterisks (**) indicate a significant correlation at *p* ≤ 0.01, and Three asterisks (***) indicate *p* ≤ 0.001, significant correlation.

The pH and organic matter of the collected banana rhizosphere soil were analyzed. The pH of the banana planting soil was acidic, ranging between 3.6 and 6.5. Under normal circumstances, the soil organic matter content of the tillage layer is usually above 50 g/kg soil, but in the banana rhizosphere soil, this averaged 30 g/kg soil, which is 40% lower than the normal level. The correlation between soil pH, organic matter and soil microorganisms in the banana rhizosphere at the genus level was analyzed. The bacterial genera as a whole showed a significant negative correlation with pH value; only the bacterial genera uncultured_bacterium_c_*Subgroup*_6 and *Gemmatimonas* showed a significant positive correlation with pH value, with the genus uncultured_bacterium_c_*Subgroup*_6 having a stronger correlation with pH. The fungal genera as a whole showed no significant negative correlation with pH; only the fungal genus *Coprinellus* showed a positive correlation with pH value, but it was not significant. The negative correlation between bacterial genera and pH value was higher than that of the fungal genera, and acidic pH had a greater impact on bacteria than on fungi. For the soil environment with lower organic matter levels, most bacterial genera showed no correlation with lower organic matter in the soil environment; only the bacterial genera *Bradyrhizobium* and uncultured_bacterium_f_*Xanthobacteraceae* showed a low, positive correlation with organic matter level. Some fungal genera were negatively correlated, with organic matter level, but most were not correlated. The fungal genus *Fusarium* showed a weak positive correlation with the current pH and organic matter level, but the correlation was not significant (Fig. 6).

### Correlation analysis between metabolites and microorganisms in banana rhizosphere soil

**Figure 7:**
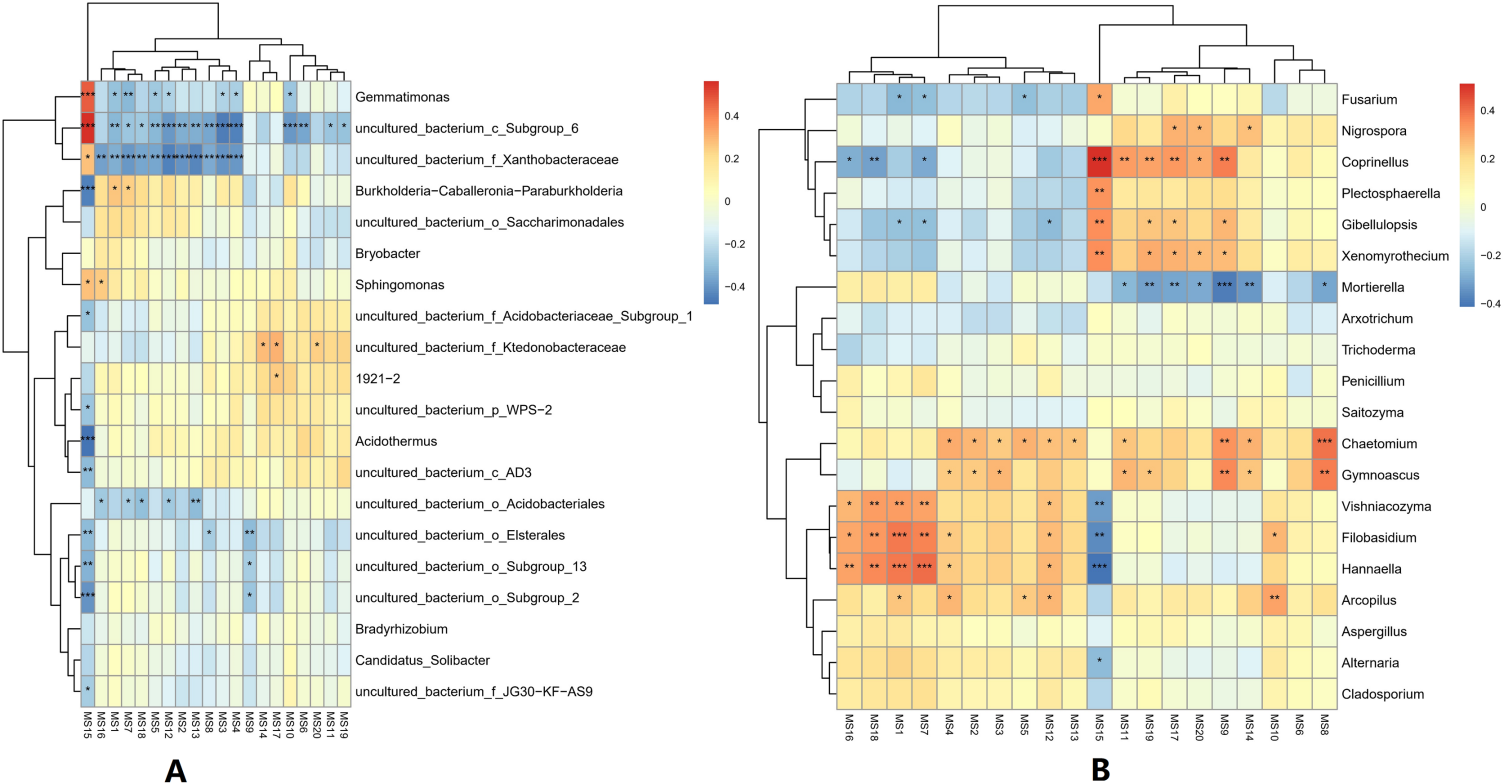
Correlation analysis between metabolites and microorganisms in banana rhizosphere soil. (A) Heatmap of correlations between bacterial genera and metabolites. (B) Heatmap of correlations between fungal genera and metabolites**. **The horizontal axis is the different metabolites and the vertical axis is the species of microorganisms at the genus level. Color intensity indicates the level of correlation; red represents a positive correlation, blue represents a negative correlation. An asterisk (*) indicates a significant correlation at *p* ≤ 0.05, Two asterisks (**) indicate a significant correlation at *p* ≤ 0.01, and Three asterisks (***) indicate a significant correlation at *p* ≤ 0.001. MS1: UDP-D-galacto-1,4-furanose; MS2: dIDP; MS3: ribavirin-5′-monophosphate; MS4: L-Ascorbate-6-phosphate; MS5: thiamine thiothiazolone pyrophosphate; MS6: cis-4-Carboxymethylenebut-2-en-4-olide; MS7: UDP-Glucuronic acid; MS8: NAIR; MS9: Sulfamethizole; MS10: P1-uridyl-P2-phenyl diphosphate; MS11: 2′, 3′-Cyclic UMP; MS12: sn-glycerol-1-phosphate; MS13: 3-phosphoglycerate; MS14: D-Erythro-imidazole-glycerol-phosphate; MS15: 2-Chloro-L-phenylalanine; MS16: CDP; MS17: Tartronate semialdehyde; MS18: P1-uridyl-P2-methyl diphosphate; MS19: D-Ribulose-5-phosphate; MS20: N-carbamoyl-L-aspartate.

At the genus classification level, the correlations between the top 20 metabolites and microorganisms in the soil were analyzed. For bacterial genera, the 2-Chloro-L-*phenylalanine* metabolite showed a significant positive correlation with bacteria uncultured_bacterium_c_Subgroup_6, *Gemmatimonas*, and a significant negative correlation with *Burkholderia*-*Caballeronia*-*Paraburkholderia*, and *Acidothermus*, while bacteria uncultured_bacterium_c_Subgroup_6 and uncultured_bacterium_f_Xanthobacteraceae showed a significant positive correlation with most of the metabolites. Acidothermus have a negative correlation with metabolites except for 2-Chloro-L-phenylalanine. For the fungal genera, the 2-Chloro-L-phenylalanine metabolite showed a significant positive correlation with the fungal genera *Coprinellus*; it also showed a strong positive correlation with the fungal genera *Plectosphaerella*, *Gibellulopsis*, and *Xenomyrothecium*, significant negative correlation with the fungal genera *Hannaella*, and a strong negative correlation with the fungal genera *Vishniacozyma* and *Filobasidium*. In addition, the UDP-D-galacto-1, 4-furanose and UDP-Glucuronic acid metabolites showed strong positive correlation with the fungal, *Filobasidium* and *Hannaella*, respectively. There was also a strong positive correlation between the NAIR metabolite and fungal *Chaetomium* and *Gymnoascus*. A strong negative correlation was seen between the fungal genus *Mortierella* and the D-Ribulose 5-phosphate, Tartronate semialdehyde, Sulfamethizole, and D-Erythro-imidazole-glycerol-phosphate metabolites, and between the P1-uridyl-P2-methyl diphosphate metabolites and the fungal genus *Coprrinellus*. Overall, fungal genera and metabolites exhibited more complex correlation networks than bacterial genera (Fig. 7).

### Network relationship analysis among banana rhizosphere soil microorganisms

**Figure 8:**
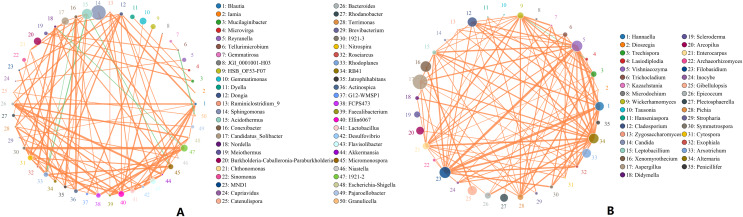
Analysis of inter-bacterial and inter-fungal networks in banana rhizosphere soils at the genus level. (A) The correlation network of each species at the bacterial genus level. (B) Correlation network diagram of each species at the fungal genus level. The circle represents the species and the size of the circle represents the average abundance of the species. The lines represent the correlation between two species; the thickness of the line represents the strength of the correlation, and the color of the line represents the type of correlation: orange represents a positive correlation, green represents a negative correlation.

**Figure 9:**
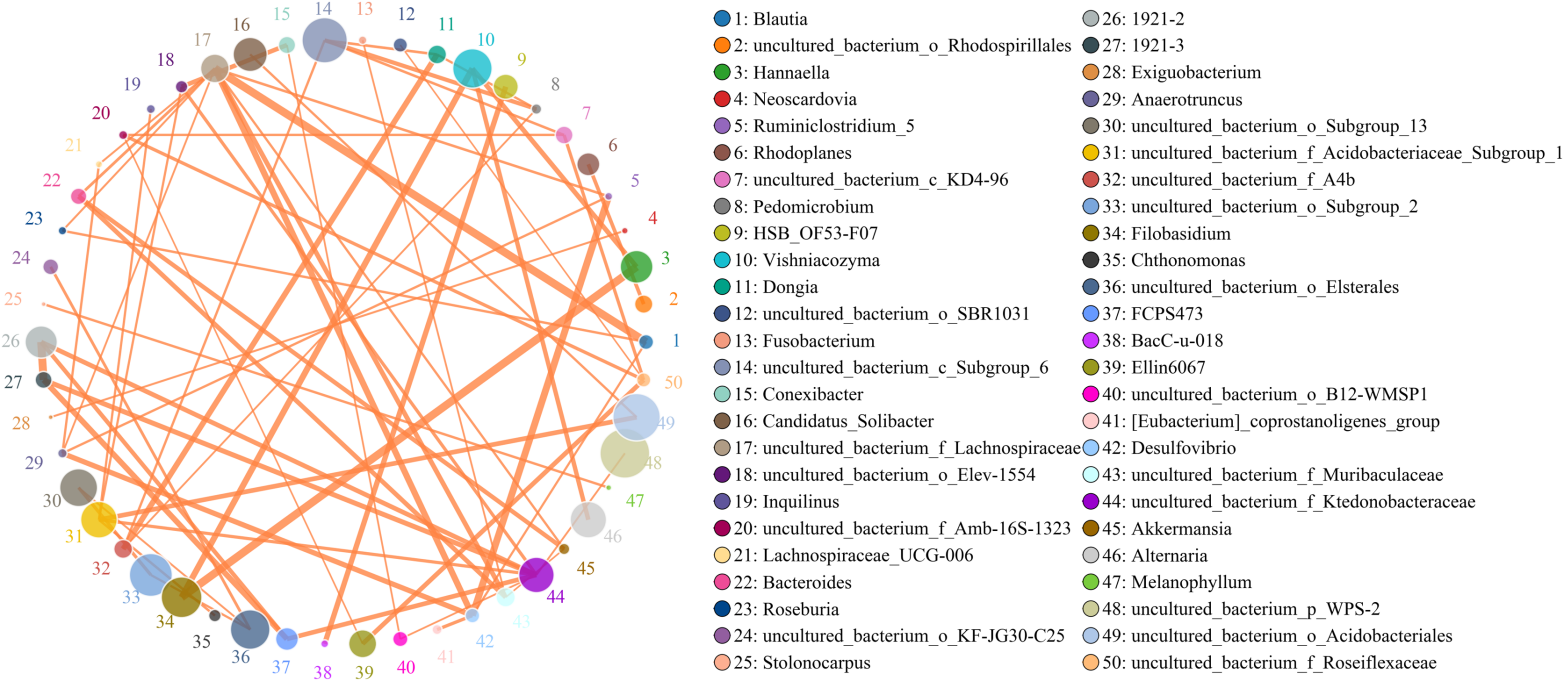
Analysis of the network relationship between soil microorganisms (bacteria and fungi) in banana rhizosphere soils at the genus level. The circle represents the species and the size of the circle represents the average abundance of the species. The lines represent the correlation between two species, the thickness of the line represents the strength of the correlation, and the color of the line represents the type of correlation: orange represents a positive correlation.

Microbes are considered mutualistic, symbiotic, or pathogenic, and the microbial diversity of banana rhizosphere soils is intricate. The network relationship of each species was studied at the bacterial genus level, and it was found that the network interaction relationship between most bacterial genera showed positive correlation, and the network interaction relationship between microorganisms from the same of genera was stronger (for example: between unspecified bacterial genera 1921–2 and 1921–3). There are few negative correlations in bacterial genera, including between bacterial genera *Acidothermus* and bacterial genera *Rhodoplanes* and *Micromonospora*; between *Rhodoplanes* and *Conexibacter*; between *Nordella* and 1921–2; and between *Tellurimicrobium* and *Granulicella*. The network relationship of fungal genera species was also mostly positive, which is similar to that of bacteria. The fungal genus *Filobasidium* had the strongest positive correlation with *Vishniacozyma* and *Hannaella*. Interestingly, the smaller the average abundance of the bacterial genera, the more complex its positive correlation network is with other bacterial genera; while the opposite was observed for fungal genera: the larger the levels of the fungal genera species, the more complex its positive correlation network is with other fungal genera (Fig. 8). At the genus level, bacterial genera and fungal genera were mainly positively correlated, which also indicated that the relationship between bacteria and fungi in the rhizosphere of healthy banana soil was mainly one of mutual promotion (Fig. 9).

## Discussion

Rhizosphere microorganisms are crucial to the growth and development of crops, and they interact and promote each other within the root system of crops. There is no current research that analyzes the microbial diversity background of healthy banana rhizosphere soil, so this study carried out an analysis of the microbial composition of healthy banana rhizosphere soil during one growth cycle. Studies have shown that Proteobacteria and Acidobacteria are also major microbial players in most soils (Bahram et al., 2018; Liu, Ding & Wang, 2020), and our findings are similar. Proteobacteria, Chloroflexi and Acidobacteria dominated in the healthy banana rhizosphere soil. Studies have also shown that Proteobacteria is positively correlated with fusarium wilt in banana plants and negatively correlated with Acidobacteria (Koberl et al., 2017; Shen et al., 2015). Some researchers have also suggested using Proteobacteria and Acidobacteria as bacterial indicators of changes in soil environmental factors related to land use changes. Kim et al. (2021) also reported similar relative abundances of Proteobacteria and Acidobacteria in farmland soil, while the relative abundance of Acidobacteria in forest soil was about 1.8 times higher than that in farmland soil samples, and the relative abundance of Proteobacteria was slightly higher than that in farmland soil. They argue that a change in land use from forest to cropland, where soils are treated with cow dung and pesticides, results in higher abundances of bacterial members of Proteobacteria, but a decreasing trend in the relative abundance of Acidobacteria (Kim et al., 2021). The current study found that the fungal taxa Ascomycota was the most abundant in healthy banana rhizosphere soil. Ascomycota is morphologically diverse and includes unicellular yeast, filamentous fungi, and more complex goblet fungi. Shen et al. (2015) compared the differences in the rhizosphere flora of bananas using bio-organic fertilizers and chemical fertilizers for 2 years, and the results showed that the relative abundance of Ascomycota in soils using organic fertilizers was significantly lower than in the groups using chemical fertilizers. They also showed that Ascomycota was positively correlated with fusarium wilt in banana plants (Shen et al., 2015). Therefore, an increase in bacterial phylum Acidobacteria levels and a decrease in the relative abundances of the bacterial phylum Proteobacteria and the fungal phylum Ascomycota can indicate healthy banana growth and a low probability of banana wilt.

From the genus level of microbial taxonomy, our results found that the bacterial and fungal genera in the rhizosphere of healthy banana soil are both widely distributed and diverse. We also found that the relative abundance of each bacterial genus was lower in the rhizosphere of healthy banana soils, which may be the result of microbial competition. Uncultivated bacterial genera accounted for the largest portion of the bacterial genera in healthy banana soil, and among the culturable bacterial genera, *Sphingomonas* had the highest abundance, indicating that uncultivated microorganisms also contributed significantly to the healthy growth of banana plants. The experimental results of Shen et al. (2015) also showed that the bacterial genus *Sphingomonas* was relatively abundant in banana soil, but their experimental results also showed that *Dokdonella*, *Sphingobium*, *Flavobacterium*, *Mesorhizobium*, *Chryseobacterium*, *Sphingomonas*, *Dyadobacter*, and *Devosia* occupy a relatively high relative abundance in banana soil. We detected a higher abundance of uncultured bacterial genera in banana soil, so our experimental results differed from those of Shen et al. (2015). Zhou et al. (2019) compared disease-free soils, and the results showed that the relative abundance of *Sphingomonas* in disease-free soils was higher than that in diseased soils. The main fungal genera in banana soil are *Mortierella*, *Fusarium*, and *Penicillium*; *Fusarium* is the pathogen that causes banana fusarium wilt in banana plants. The results of Zhou et al. (2019) also indicated that *Fusarium*, *Pseudallescheria* and *Nectriaceae* were enriched in banana wilt soil and were lower in disease-free soil. *Fusarium* had a relative abundance of 16.39% in banana wilt soil; *Fusarium* was also detected in healthy, disease-free soil, but with a lower relative abundance of 8.30% (Zhou et al., 2019). The relative abundance of *Fusarium* in healthy banana soils in this study varied from 4.37% to 12.50%, with an annual average relative abundance of 7.80%. This further shows that the presence of *Fusarium* in banana soil does not necessarily lead to the occurrence of Fusarium wilt. The occurrence of banana fusarium wilt may be caused by the imbalance of microbial flora in banana soil, resulting in the enrichment of *Fusarium*, increasing its relative abundance above healthy levels. In addition, the results of Zhou et al. (2019) also found that the relative abundances of *Mortierella* and *Penicillium* were significantly higher in healthy banana soils than in diseased soils. The main fungal genera in the healthy banana soil in this study were *Mortierella*, *Fusarium* and *Penicillium*. An increase in the relative abundance of Fusarium leads to a decrease in the relative abundance of *Mortierella* and *Penicillium* as *Fusarium* competes with *Mortierella* and *Penicillium*. Our bacterial genera and fungal genera results were similar, but *Pseudallescheria* and *Nectriaceae* were not found in our soil.

The differences in the Shannon indices of banana soil bacteria and fungi were not significant in any growth stage, indicating that the diversity of banana soil microecology is in an overall stable state, and the small fluctuations seen in each growth period are steady state dynamic fluctuations. Throughout the entire planting cycle of bananas, bacterial diversity remains stable, while the fungal diversity fluctuates greatly. As growth time increased, bacterial *Proteobacteria* showed a gradual decline at first and then slightly fluctuated up and down with the lowest relative abundance in the M4 growth period, while *Acidobacteria* was relatively stable, with the relative abundance only slightly fluctuating around 14.41%. The fungal phylum *Ascomycota* first showed a large decline in the M2 period, then a rapid increase in the M3 period, and then a dynamic change of a slow increase followed by a slow decline. The relative abundance of fungal phylum *Basidiomycota* declined the most in the M2 and M6 growth periods, and fluctuated less in the other periods. The relative abundance of fungal phylum *Mortierellomycota* increased sharply in the M2 period, then only slightly fluctuated in the other growth periods. Bacterial genus *Sphingomonas* was the lowest in the M1 stage, and then maintained its average relative abundance in other stages. The genus *Mortierella* showed a sudden surge in the M2 phase and then dropped sharply to a lower level. The relative abundance of *Fusarium* continued to increase with time, reaching its highest level in the M6 growth stage before starting to decline again in the M7 stage. The relative abundance of *Penicillium* sharply decreased in the M2 stage, and then gradually decreased after a brief increase in the M3 stage. The drastic changes in the M2 period may be related to the climate of the M2 period. The M2 growth period is the driest month of the year, but since the banana plant is only 2 months old, it is still small, so the soil surface area shaded by leaves is also small, leading to a significant loss in soil moisture. Most fungi prefer an environment with abundant rainfall and a humid climate, so the M2 stage is not an ideal growth stage for most fungi, but *Mortierella* may be more drought-resistant in these stages and gain growth advantages. Bahram et al. (2018) showed that fungi and bacteria exhibit global niche differentiation in relation to precipitation.

Metabolic profiles in rhizosphere soils include a variety of chemicals that recruit specific microbial species to form complex relationships with plants (Kuzyakov & Razavi, 2019), and the chemical classes found in the root soils of different plants are highly diverse, consisting mainly of organic acids followed by sugars and their derivatives (Carvalhais et al., 2010; Liu, Ding & Wang, 2020). We analyzed the rhizosphere metabolites of healthy banana plants and found that the most common metabolites were sugars, lipids and organic acids, accounting for about 50% of the total metabolites, which was similar to the results of previous studies. We detected a total of 130 metabolites in banana rhizosphere soil, including 17 sugars and their derivatives, 34 organic acids, eight bases and nucleosides, 18 esters, eight alcohols and aldehydes, 13 amino acids, nine insecticides and biotin, six kinds of amines and derivatives, and five kinds of aromatic compounds. The differences in the metabolites present in each growth stage were not significant, indicating that most of the metabolites in the soil were stable. Most of the metabolites present were nutrients required for the growth of banana plants and soil microorganisms, which may be the result of microbial competition as well as mutually beneficial microbial relationships in the banana rhizosphere soil. Some studies have found that organic acids and sugars can be preferentially used as nutrients by certain bacteria, enhancing the acquisition of organic matter and phosphorus by crops, promoting the formation of some rhizosphere bacterial biofilms, and releasing toxic elements from the soil (Cheng, Zhang & He, 2019; Vause, Heaney & Lin, 2018; Voges et al., 2019). Metabolomics technology allows us to better understand the composition of metabolites in the rhizosphere, but the current metabolomic database is incomplete and in need of further development and improvement.

There is an intricate network relationships between microorganisms and the environment in banana rhizosphere soil. To better understand these relationships, we analyzed the relationship between microorganisms and pH, organic matter and rhizosphere metabolites, and microorganisms and rhizosphere metabolites through microbial taxonomy. In a soil environment with an acidic pH value, the bacterial genera as a whole showed a significant negative correlation with pH, and the fungal genera showed an insignificant negative correlation, indicating that the acidic soil environment gave rhizosphere bacteria a competitive advantage. If the soil remains acidic, however, bacterial diversity may decrease as previous studies have shown that the competition for rhizosphere bacteria that can survive in soils with pH < 5.5 may be relatively small (Wan et al., 2020). Previous studies have shown that bacterial and fungal communities are mainly driven by soil organic matter (Shen et al., 2018), but the organic matter level of the banana rhizosphere soil in this study was low, and most bacterial genera showed no correlation; only the bacterial genera *Bradyrhizobium* and uncultured_bacterium_f_*Xanthobacteraceae* showed a low, positive correlation with organic matter level. Fungal *Mortierella* showed a significant positive correlation and Chaetomium showed a significant negative correlation with organic matter level. Although the banana plants were still healthy at these levels in our study, without supplementing the organic matter, fusarium wilt would likely occur eventually. Previous studies have shown that soil organic matter is significantly negatively correlated with banana fusarium wilt incidence and *Fusarium* abundance, suggesting its potential role in disease suppression (Shen et al., 2018). While the fungal genus *Fusarium* found in this study was significantly correlated with current pH, it only had a weak positive correlation with organic matter which was not significant, indicating that although the banana plants could still grow in this study plot, as pH and organic matter continued to decrease, the risk of fusarium wilt would increase. Comparing the correlation between bacterial and fungal genera for lower soil organic matter levels, fungal genera showed both positive and negative correlations to lower soil organic matter levels, but most were not significantly correlated. Conversely, because bacteria levels were low, bacterial genera levels only had to change slightly to be significantly correlated with low soil organic levels, so the significant correlations in multiple bacterial genera. The 2-Chloro-L-phenylalanine metabolite showed a significant positive correlation with bacteria uncultured_bacterium_c_*Subgroup*_6 and *Gemmatimonas*, a significant negative correlation with *Burkholderia*-*Caballeronia*-*Paraburkholderia*, *Acidothermus*, and with fungal genera *Hannaella*, *Vishniacozyma*, and *Filobasidium*, a significant positive correlation with the fungal genera *Coprinellus*, *Plectosphaerella* and *Gibellulopsis*, and a strong positive correlation with *Xenomyrothecium*. *Acidothermus* showed no correlation with other metabolites. In addition, the metabolites UDP-D-galacto-1, 4-furanose and UDP-Glucuronic acid showed strong positive correlations with fungal, *Filobasidium* and *Hannaella*, and between the NAIR metabolite and the fungal, *Chaetomium* and *Gymnoascus*. There were strong negative correlations between the fungal genus *Mortierella* and the D-Ribulose 5-phosphate, Tartronate semialdehyde, Sulfamethizole, and D-Erythro-imidazole-glycerol-phosphate metabolites, and between the P1-uridyl-P2-methyl diphosphate metabolites and the fungal genus *Coprinellus*. In general, fungi are sensitive to banana rhizosphere soil metabolism, and the correlation network between fungi and metabolites was more complex than that of bacteria. Previous studies have also shown that most metabolites are positively associated with some key taxa and negatively associated with others. This may be due to microbial metabolic differentiation and resource constraints, as different microorganisms have different preferences for the same substrate (Zhalnina et al., 2018). Microbial networks are often composed of many symbiotic components networked in parasitic, symbiotic, non-symbiotic, or synergistic modes, and these exchanges may affect the suitability of each component, with direct effects on soil fertility and plant health (Babalola et al., 2020; Fierer, 2017). This study found that the overall network interaction between bacterial genera was positively correlated, and the network interaction between microorganisms from similar genera was stronger (for example: unspecified bacterial genera 1921–2 and 1921–3). There were a few negative correlations in bacterial genera, including the *Acidothermus* and *Rhodoplanes*; *Acidothermus* and *Micromonospora*; *Rhodoplanes* and *Conexibacter*; *Nordella* and 1921–2; and *Tellurimicrobium* and *Granulicella*. The network relationship of fungal species was also mostly positive, similar to that of bacteria. The fungal genus *Filobasidium* had the strongest positive correlation with *Vishniacozyma* and *Hannaella*. The relationship between bacterial and fungal genera was mainly positive, indicating that the relationship between bacteria and fungi in the rhizosphere of healthy banana soil was mainly one of mutual promotion. This also shows that in the healthy banana rhizosphere soil, the relationships between the microorganisms are mutually beneficial, not competitive. Interestingly, the smaller the level of the bacterial genera, the more complex its positive correlation network was with other bacterial genera, while the opposite was true for fungal genera. It may be that hegemonism in bacteria is not conducive to the mutual benefit of bacteria, while fungi grow slowly and require collective hegemony to gain a growth advantage. This also suggests that in banana planting, inhibiting the pathogenic bacteria of banana fusarium wilt and promoting the overall micro-ecological network balance of the rhizosphere should both be considered before the application of microbial inoculants.

## Conclusions

Healthy banana rhizosphere soil has stable microecology and metabolic characteristics. Acidic soil environments have a strong inhibitory effect on bacterial growth, but less inhibitory effect on fungi. 2-Chloro-L-phenylalanine is a metabolite that largely impacts banana rhizosphere soil microorganisms. In healthy banana rhizosphere soil, the microorganisms have mutually beneficial, non-competitive relationships. The use of microbial inoculants in agriculture should be considered only after inhibiting the growth of pathogenic bacteria and paying attention to the overall microecological balance of the soil.

## Supplemental Information

10.7717/peerj.14404/supp-1 Supplemental Information 1 pH and organic matter of 70 soil samples. pH-1, pH-2 and pH-3 represent three replicates of pH, respectively. Organic-1, Organic-2 and Organic-3 represent the three repeats of Organic.

10.7717/peerj.14404/supp-2 Supplemental Information 2 Raw data for metabolome assays.

## Additional Information and Declarations

### DNA Deposition

The following information was supplied regarding the deposition of DNA sequences: Data is available at the Sequence Read Archive (SRA) database: PRJNA834605.

### Data Availability

The following information was supplied regarding data availability: The raw data is available in the Supplemental Files.
